# The Ketogenic Diet Increases In Vivo Glutathione Levels in Patients with Epilepsy

**DOI:** 10.3390/metabo10120504

**Published:** 2020-12-10

**Authors:** Antonio Napolitano, Daniela Longo, Martina Lucignani, Luca Pasquini, Maria Camilla Rossi-Espagnet, Giulia Lucignani, Arianna Maiorana, Domenica Elia, Paola De Liso, Carlo Dionisi-Vici, Raffaella Cusmai

**Affiliations:** 1 Medical Physics Department, Bambino Gesù Children’s Hospital IRCCS, 00165 Rome, Italy; martina.lucignani@opbg.net; 2 Neuroradiology Unit, Bambino Gesù Children’s Hospital IRCCS, 00165 Rome, Italy; daniela.longo@opbg.net (D.L.); lucapasquini3@gmail.com (L.P.); mcamilla.rossi@opbg.net (M.C.R.-E.); giulia.lucignani@opbg.net (G.L.); 3 Nesmos Department, Sapienza University, 00165 Rome, Italy; 4 Division of Metabolic Diseases, Bambino Gesù Children’s Hospital IRCCS, 00165 Rome, Italy; arianna.maiorana@opbg.net (A.M.); carlo.dionisivici@opbg.net (C.D.-V.); 5 Artificial Nutrition Unit, Bambino Gesù Children’s Hospital IRCCS, 00165 Rome, Italy; domenica.elia@opbg.net; 6 Child Neurology Unit, Bambino Gesù Children’s Hospital IRCCS, 00165 Rome, Italy; paola.deliso@opbg.net (P.D.L.); raffaella.cusmai@opbg.net (R.C.)

**Keywords:** ketogenic diet, magnetic resonance spectroscopy, glutathione

## Abstract

The Ketogenic Diet (KD) is a high-fat, low-carbohydrate diet that has been utilized as the first line treatment for contrasting intractable epilepsy. It is responsible for the presence of ketone bodies in blood, whose neuroprotective effect has been widely shown in recent years but remains unclear. Since glutathione (GSH) is implicated in oxidation-reduction reactions, our aim was to monitor the effects of KD on GSH brain levels by means of magnetic resonance spectroscopy (MRS). MRS was acquired from 16 KD patients and seven age-matched Healthy Controls (HC). We estimated metabolite concentrations with linear combination model (LCModel), assessing differences between KD and HC with *t*-test. Pearson was used to investigate GHS correlations with blood serum 3-B-Hydroxybutyrate (3HB) concentrations and with number of weekly epileptic seizures. The results have shown higher levels of brain GSH for KD patients (2.5 ± 0.5 mM) compared to HC (2.0 ± 0.5 mM). Both blood serum 3HB and number of seizures did not correlate with GSH concentration. The present study showed a significant increase in GSH in the brain of epileptic children treated with KD, reproducing for the first time in humans what was previously observed in animal studies. Our results may suggest a pivotal role of GSH in the antioxidant neuroprotective effect of KD in the human brain.

## 1. Introduction

Ketogenic Diet (KD) is a high-fat, low-carbohydrate diet that has been utilized as an adjuvant treatment for epilepsy for over 90 years [1] KD mimics the effect of fasting, causing the body to maintain a prolonged state of ketosis. The first event occurring during KD is the blood level increase of three ‘ketone bodies’, namely Acetoacetate (AcAc), 3-B-Hydroxybutyrate (3HB) and Acetone. AcAc accumulates during enhanced fatty acid metabolism under low carbohydrate conditions, while 3HB is formed from the reduction in AcAc within the mitochondria. These two predominant ketone bodies are energy-enriched compounds that transport energy from the liver to other tissues. Over the years, KD has become an elective indication for contrasting intractable epilepsy. Brain tissue is vulnerable to oxidative damage because of its high oxygen consumption, with consequent generation of high levels of reactive oxygen species (ROS). ROS have been associated with damage to cerebral tissue in several neurological diseases, such as epilepsy itself. Ketosis induced by KD elevates the anti-oxidative capacity of the central nervous system in animal models [2] and improves patient conditions [3]. The mechanism of action of KD is heterogeneous, including the shifting of glycolysis products towards lipid metabolism, and the regulation of mitochondrial metabolism. Moreover, KD may regulate neuronal activity and transmission through different mechanisms, influencing neurotransmitter balance and gene expression [4,5]. The neuroprotective effect of ketone bodies has been widely shown in recent years [2,6,7,8,9,10,11], but the mechanism underlying the influence of KD on mitochondrial redox status has yet to be fully understood. Furthermore, very little is known of the in vivo metabolic dynamics of KD and how antioxidant activity might be triggered by a high level of ketone bodies in humans. Glutathione (GSH) is a tripeptide thiol found virtually in all cells. Among its many roles, it is implicated in oxidation-reduction reactions, acts as an enzyme cofactor, and protects against ROS [11,12,13]. Remarkably, a previous study has shown that epileptic seizures lead to a depletion of mitochondrial GSH [14], supporting the idea of a link between epilepsy and oxidative stress, with possible beneficial effects of KD in this condition. Few animal studies demonstrated that GSH or GSH-related enzymes increase during KD, thus suggesting a possible role for GSH as a ‘biomarker’ of good redox status [2,15]. Nevertheless, no evidence of such an increase has been observed in vivo in the human brain to date. Our aim is to estimate brain GSH levels in patients undergoing KD adjuvant therapy by magnetic resonance spectroscopy (MRS) to investigate whether GSH increases are detectable compared to healthy controls (HC), in a similar way to animal studies.

## 2. Results

**Figure 1:**
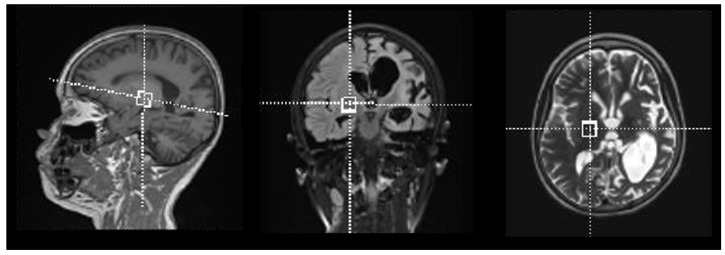
Voxel positioning within right ganglio-capsular region.

**Figure 2:**
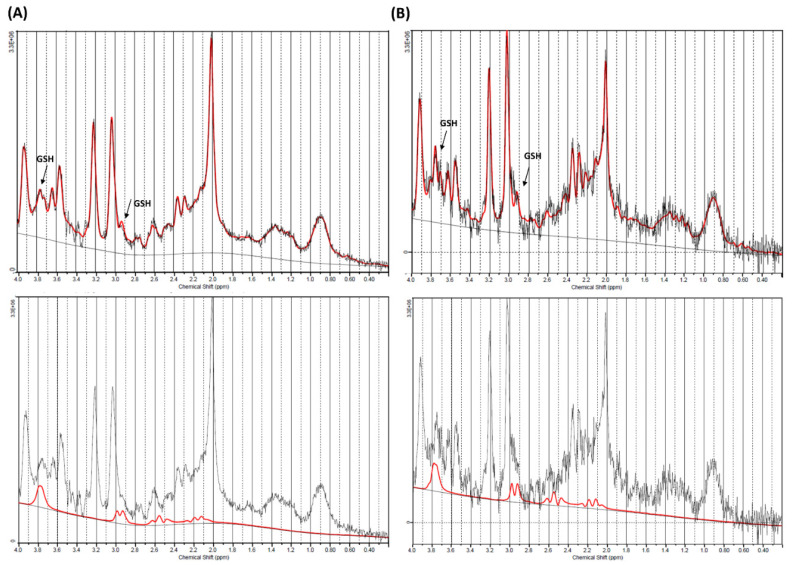
Magnetic resonance (MR)-spectrum obtained within ganglio-capsular volume of interest (VOI) for a control (**A**) and a Ketogenic Diet (KD) patient (**B**). Composite spectrum (above) presents glutathione (GSH) peaks of approximately 2.95 and and 3.75 ppm (black arrow), that are more visible on the GSH fitting spectra (below).

Voxels were located in the ganglio-capsular region of the right hemisphere (Figure 1). For each patient, GSH absolute concentrations and GSH concentration relative to creatine/phosphocreatine (GSH/Cr) are reported in Table 1, along with CRLB, n-acetylaspartate (NAA)-to-creatine ratio (NAA/Cr), linewidth and Signal to noise ratio. The quality of spectra was good for most of the study subjects, with only one patient excluded from the statistical analysis as due to incorrect water suppression. Figure 2 reported the composite spectrum from the ganglio-capsular VOI with the LC Model fit overlaid for a control (Figure 2A) and a KD patient (Figure 2B). Mean concentrations of GSH (KD: 2.5 ± 0.5 mM, HC: 2.0 ± 0.5 mM) show a 15% GSH increase in the KD group compared to HC (*p* < 0.04). Pearson analysis revealed no correlation between GSH and blood serum 3HB concentrations. Similarly, no correlation was observed between GSH and number of seizures.

## 3. Discussion

The present study employs in vivo MRS to monitor KD effects on brain metabolism. In particular, we focused on GSH to investigate concentration changes in this important metabolite following KD therapy. According to our results, patients undergoing KD showed higher levels of brain GSH compared to HC. Jarret et al. reported higher GSH concentration levels in the hippocampal mitochondria of rats under KD compared to controls by using High Performance Liquid Chromatography [15]. Although the evidence of high GSH levels was reported in the hippocampus alone, such a result is quite in line with our findings suggesting that KD might stimulate de novo GSH synthesis and improve the redox status of the brain. It is indeed well established that GSH plays a neuroprotective role against oxidative stress, with several diseases originating from its dysregulation [16,17,18]. GSH is a product of glycolysis, able to modulate glutamate receptors and calcium influx [19]. A GSH increase was also demonstrated in a Parkinson’s disease animal model. The authors reported elevated GSH levels in KD-fed rats compared to controls [20], validating the idea that ketone bodies may influence GSH concentration. The mechanism underlying GSH increase during KD may involve the upregulation of nuclear factor erythroid 2-related factor 2 (NRF2) transcription factor, a primary responder to cellular stress which promotes GSH biosynthesis, as demonstrated in rats [4]. The upregulation of Nrf2 may depends on the mild oxidative and electrophilic stress initially induced by the KD, leading to chronic cellular adaptation, induction of protective proteins, and stable improvement in redox state [4]. All known brain antioxidants, directly or indirectly, depend on NADPH production, and 3HB is implicated in elevating the levels of NADH producing AcAc [21]. Moreover, among all ketone bodies, 3HB seems to be involved in decreasing the NADP^+^/NADPH ratio through a variety of mechanisms such as decreasing the glycolytic flux [22,23,24,25] or increasing the concentration of mitochondrial acetyl-CoA [22]. There is also a tight link between NADH and NADPH, as the latter is synthetized via nicotinamide nucleotide transhydrogenase by transferring hydride ions from NADH to NADP^+^ [23,25]. High levels of NADPH translate into an increase in several antioxidants [26]: GSH is surely one of them, since it participates in the chemical equation

(1) $$GSSG+NADPH\;\leftarrow \to 2\;x\;GSH+NADP^{+}$$

where GSSG is glutathione disulfide, the oxidized form of glutathione [13]. Moreover, 3HB is also an endogenous inhibitor of class I histone deacetylases (HDACs), which are epigenome modifiers that act on chromatin by removing acetyl groups from histone tails. This process is known to impact cells’ oxidative balance by inducing protector genes such as Foxo3 and Mt2 [27,28]. In conflict with our results, an in vitro study showed that ketone bodies may reduce reactive oxygen species without GSH increase [8]. This discrepancy might depend on GSH homeostasis impairment due to the particular experimental setting for the in vitro study. On the other hand, no correlation was observed between blood 3HB and GSH levels in our study. The reason for this might rely on differences in medications, lifestyle and age among our patients, which may influence GSH levels. Even though a study on epileptic patients showed a reduction in GSH as due to the effect of seizures [14], our study failed to observe a correlation between GSH and number of seizures. Multiple factors can affect patient response to KD, leading to a GSH level misbalance in cases of epileptic seizure [29]. Furthermore, patient response may vary on a genetic basis [29].

Our study has an intrinsic limitation due to the small number of patients included. However, KD is a very specific adjuvant treatment reserved for few rare clinical conditions, so large populations are difficult to recruit. In addition, there is no clear evidence about the possible effects of other medications on GSH levels. Anesthesia might be a further possible confound for the present study, as it could modify the GSH brain [30]. However, sevoflurane was the only anesthetic employed in the study, which has been shown to reduce GSH levels rather than increase them [30]. Consequently, we can hypothesize the anesthesia-confounding effect as being negligible. From a methodological perspective, GSH is often quantified using a J-edited MEGAPRESS sequence with a longer echo time (TE), which demonstrated reliable estimation of GSH concentrations [31], as well as a better performance with time domain fitting methods [32]. Due to time constraints related to the clinical practice, our protocol was rather based on a short echo time PRESS sequence. Such a protocol has been widely used in a variety of disorders to quantify GSH [33,34,35,36,37,38,39,40] and was validated several times with phantom studies [33,34], by assessing reproducibility [39] and by comparing the results with 7T quantification [38]. Further studies are needed to exploit MEGA-PRESS clinical applications, including the effects of extended and closed GSH forms in the brain under KD, similarly to what is performed in Alzheimer disease [41]. Future investigation on ketone bodies’ concentration before and during KD will further develop our understanding of the effects of this therapy, especially if compared to epileptic patients with different treatments.

## 4. Materials and Methods

### 4.1. Standard Protocol Approvals, Registrations, and Patient Consents

The study has been approved by our IRB with waiver of informed consent due to retrospective design. Specific informed consent for the execution of MR examinations was obtained from every patient/next of kin. The study was carried out in agreement with the principles of the Helsinki declaration. This research was approved by the ethical committee of Bambino Gesù Children's Hospital (protocol number 1867/2019).

### 4.2. Subjects

We identified 31 patients treated with classic KD. Each patient followed a diet with a specific KD ratio (ratio of lipids to combined fat and protein), depending on clinical course and seizure response. Out of 31 subjects, 17 KD patients (mean age = 7.3 y, range = 0.4−16 y, 50% female) underwent MRS during their treatment, producing 17 spectra. Additionally, a group of seven age-matched Healthy Controls (HC) (mean age = 9.8, range = 6−17 y, 43% female) was included in the study for comparison. A steady 3HB concentration was assured before and during fasting, soon before and soon after the end of the procedure. Clinical data on patient age, diagnosis, medications, diet duration and seizure occurrence (mean number of seizures per week computed over a period of 4 weeks) were reported in Table 1.

### 4.3. Data Acquisition

Brain MRI and MRS were acquired on a 3T scanner (Siemens Magnetom Skyra, Siemens Medical Systems, Erlangen, Germany) equipped with a 32-channel head-coil. Each participant received a single voxel point resolved MRS sequence PRESS (TR: 1980 ms, TE: 30 ms, FA: 90 degrees, Number of averages: 120), with different sizes of VOI (ranging from 3 to 8 cm^3^, depending on brain dimensions) located in the ganglio-capsular region of the right hemisphere (Figure 1). The area was chosen based upon optimal shimming with similar concentrations as in cortex and for its reproducibility [40]. All examinations were performed under sedation via Sevorane. All the acquisitions were performed after an automatic second order shimming on the volume of interest, and a visual inspection on the inline display was used to check for data quality. Water suppression was also applied as implemented in the Siemens platform to avoid baseline distortion. A further unsuppressed water spectrum with only 16 averages was acquired for reference purposes.

### 4.4. Data Processing

Metabolite concentrations were estimated using linear combination model (LCModel) [42]. An appropriate basis-dataset was used and water signal served as internal standard for absolute quantification. The reliability of metabolite quantification was judged upon Cramer Rao lower bounds (CRLB) as well as spectra linewidth. The standard recommended quality criterion of CRLB < 20% was applied to exclude GSH concentration values which were not reliable for the following analysis. Signal to Noise Ratio was also included as a factor for quality control.

### 4.5. Statistical Analysis

Heteroscedastic T-test was used to assess metabolite concentrations differences between KD and HC, setting the significance at 0.05. Correlations between GSH and blood serum 3HB concentrations, as well as between GSH and number of weekly epileptic seizures (see Table 1), were also investigated using Pearson’s correlation coefficient. All analyses were performed in Matlab (Matlab R2017a, The Mathworks, Inc., Natik, MA, USA).

## 5. Conclusions

The present study reproduced, for the first time in vivo and in children, what has previously been observed in animal models. Further studies are needed to verify our results, extend our findings, and clarify whether GSH changes are specific for some brain areas. However, if confirmed by further investigations, our results may suggest a protective role for KD in the brain by influencing the redox balance.

## Figures and Tables

**Table 1:** Clinical data of KD patients. Abbreviations: GSH/Cr + PCr = Glutathione to creatine/phosphocreatine ratio; NAA/Cr + PCr = N-acetylaspartate to creatine/phosphocreatine ratio; FWHM = Full width at half maximum; S/N = Signal to noise ratio; DNM1L ME = Dynamin-1-like Protein Mitochondrial Encephalopathy; PDH = Pyruvate dehydrogenase deficiency; GLUT-1 = Glucose transporter -1; GCK-HI: Congenital Hyperinsulinism caused by Glucokinase mutations; NKH = Non-ketotic hyperglycinemia; unknown EE = unknown epileptic encephalopathy; ATP1A3 = sodium-potassium ATPase catalytic subunit alpha-3; KCNT1 = potassium sodium-activated channel subfamily T member 1; PRRT2 = Proline-rich transmembrane protein 2.

KD	Age (y)	Diagnosis	Seizures (#/week)	Diet Duration (Months)	Drugs	Ketonemia (mM/L)	GSH			NAA	FWHM (ppm)	S/N
							Concentration (mM)	%SD	GSH/Cr + PCr	NAA/Cr + PCr		
1	8.9	DNM1L ME	3	39.4	topiramate, perampanel	2.0 ± 0.01	2.6	13%	0.44	0.63	0.064	5
2	3.4	PDH	28	35.7	levetiracetam, nitrazepam, allopurinol, thiamine	2.5 ± 0.01	2.8	11%	0.48	0.72	0.038	9
3	10.6	PDH	0	109.0	thiamine, carnitine, α-lipoic acid, coenzime Q, riboflavin, allopurinol	2.2 ± 0.15	2.3	12%	0.46	1.53	0.124	10
4	12.2	GLUT-1	0	49.8	allopurinol	1.8 ± 0.1	1.4	11%	0.28	1.36	0.067	21
5	16.0	GCK-HI	0	56.2	allopurinol, vitamin D	7.2 ± 0.09	2.8	9%	0.47	1.26	0.086	10
6	2.6	GLUT-1	0	21.5	multivitamin	4.4 ± 0.16	2.9	7%	0.42	0.89	0.038	12
7	4.2	NKH	28	49.0	levetiracetam, vigabatrin, levofolene, Na benzoate, dextromethorphan	2.4 ± 0.18	3.1	7%	0.38	0.53	0.093	11
8	6.7	GCK-HI	0	3.4	ethosuximide, multivitamin	3.7 ± 0.02	1.7	13%	0.44	1.32	0.029	12
9	8.5	NKH	1	98.4	levofolene, clonazepam, lansoprazole, levetiracetam, trihexyphenidyl	3.0 ± 0.01	2.6	14%	0.33	0.59	0.043	8
10	7.3	Unknown EE	21	41.9	levetiracetam, clobazam, phenobarbital	2.9 ± 0.15	3.0	9%	0.48	0.79	0.064	11
11	5.7	ATP1A3	1	46.6	valproic acid, levetiracetam	2.0 ± 0.01	2.5	10%	0.40	1.04	0.064	10
12	8.6	Chromosome 21q and 15q deletions	0	7.5	vigabatrin, valproic acid	1.7 ± 0.01	3.1	4%	0.55	1.62	0.063	47
13	6.8	Lissencephaly	14	38.3	vigabatrin, phenobarbital	2.3 ± 0.32	2.2	8%	0.46	1.31	0.024	23
14	12.4	Unknown EE	14	82.9	felbamate, clobazam	3.2 ± 0.01	2.5	8%	0.46	1.17	0.071	12
15	0.4	KCNT1	4	1.6	topiramate, levetiracetam, vigabatrin	4.4 ± 0.01	2.8	11%	0.43	0.65	0.029	10
16	2.9	PRRT2	14	13.9	vigabatrin, clobazam	1.2 ± 0.01	2.0	10%	0.29	0.83	0.057	12
